# Pd- and Ni-catalyzed cross-coupling reactions in the synthesis of organic electronic materials

**DOI:** 10.1088/1468-6996/15/4/044201

**Published:** 2014-07-07

**Authors:** Shiqing Xu, Eun Hoo Kim, Alexander Wei, Ei-ichi Negishi

**Affiliations:** Department of Chemistry, Purdue University, 560 Oval Drive, West Lafayette, IN 47907-2084, USA

**Keywords:** organic electronic materials, polythiophene, polyfluorene, polyarylene, polyarylethynylene, polyarylvinylene, cross-coupling reactions

## Abstract

Organic molecules and polymers with extended *π*-conjugation are appealing as advanced electronic materials, and have already found practical applications in thin-film transistors, light emitting diodes, and chemical sensors. Transition metal (TM)-catalyzed cross-coupling methodologies have evolved over the past four decades into one of the most powerful and versatile methods for C–C bond formation, enabling the construction of a diverse and sophisticated range of *π*-conjugated oligomers and polymers. In this review, we focus our discussion on recent synthetic developments of several important classes of *π*-conjugated systems using TM-catalyzed cross-coupling reactions, with a perspective on their utility for organic electronic materials.

## 1. Introduction

Advances in conducting and semiconducting organic materials have produced game-changing technologies for electronic devices and optoelectronic displays. The scope of organic electronic materials is extensive and cover multiple areas of technological importance: thin-film transistors (TFTs), radiofrequency identification tags, organic solar cells and light-harvesting devices, organic semiconductor lasers, and chemical sensing applications based on optoelectronic modulation [1–10]. The potential benefits of organic electronics over conventional Si-based electronics have also been stressed, such as its processability in solution, low manufacturing cost, low weight with favorable mechanical properties (e.g. flexibility and stretchability), and promise for reduced environmental impact [11, 12].

Nearly all organic electronic materials consist of *π*-conjugated backbones. Most of these are comprised of polyaromatic or heteroaromatic units; common building blocks include thiophenes, oligoacenes, fluorenes, and fused heterocyclic rings. A key contributing factor toward the tremendous advances in organic electronics is the availability of efficient synthetic methods for coupling aromatic monomers into extended *π*-conjugated systems. In particular, transition metal (TM)-catalyzed cross-coupling reactions have yielded a diverse and sophisticated class of *π*-conjugated oligomers and polymers used in organic electronics research, and TM-based catalysts with high substrate generality and turnover number continue to be developed. In this review, we focus much of our discussion on recent synthetic developments in TM-catalyzed cross-coupling reactions, with a perspective on their utility for organic electronic materials. We pay particular attention to reactions that have had a strong impact on several important classes of *π*-conjugated systems, namely oligomers and polymers of thiophenes, fluorenes, arenes and heteroarenes, arylethynylenes, and arylvinylenes.

## 2. Development of synthetic methodologies

Prior to the advent of Pd and other TM catalysts, cross-coupling reactions were limited to a handful of examples, mostly involving Grignard reagents and organoalkali species (M = Li, Na, K). Such strong nucleophiles could react with unhindered alkyl (*sp* ^3^) electrophiles in a general fashion, but their use in cross-coupling reactions between unsaturated carbon atoms (*sp* ^2^−*sp* ^2^ or *sp* ^2^−*sp* bonds) was severely limited. This problem was solved in the 1970s by the introduction of TM-catalyzed cross-coupling, which has been evolving at a rapid pace ever since. Cross-coupling methodologies were initially centered on Pd-catalyzed reactions between aryl or alkenyl halides and organometals containing Al, Zn, Zr (Negishi) [13–15], B (Suzuki) [16, 17], Sn (Stille) [18, 19], Si (Hiyama) [20, 21], and terminal alkenes (Heck) [22, 23] or alkynes (Sonogashira) [24]. Ni-catalyzed Grignard cross-couplings (Tamao−Kumada) [25, 26] (Corriu) [27] also proved to be valuable for synthesis; while more limited in scope, their efficiency could be comparable or even superior to some of the Pd-catalyzed variants mentioned above.

**Scheme 1.**
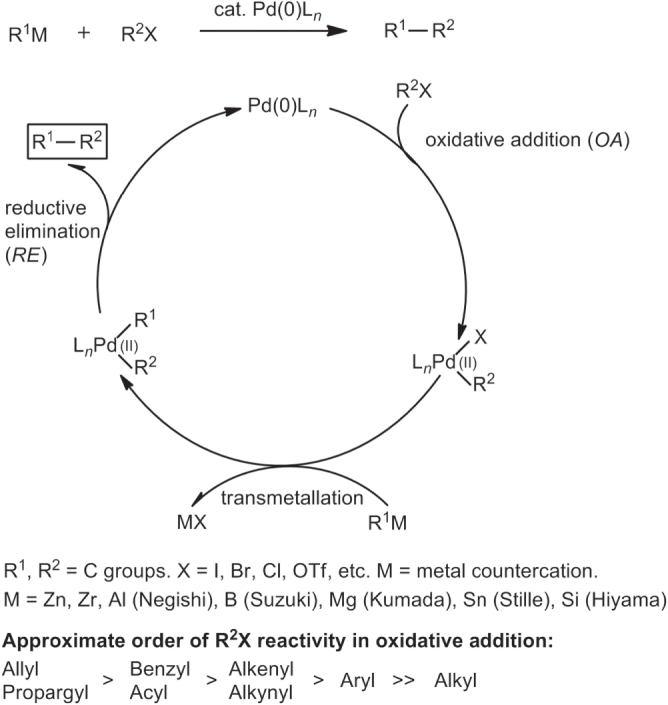
Mechanism for Pd-catalyzed C–C cross-coupling reactions.

The widely accepted mechanism for Pd-catalyzed cross-coupling reactions of organometals R^1^M with electrophiles R^2^X is depicted in scheme 1 [28]. It consists of three steps: (i) oxidative addition (*OA*) of R^2^X to Pd(0)L_*n*_ species, where L_*n*_ represents a shell of coordinating ligands, (ii) transmetallation between R^2^Pd(II)L_*n*_X and R^1^M, and (iii) reductive elimination (*RE*) of R^1^R^2^Pd(II)L_*n*_ to give R^1^R^2^ via C–C bond formation, with regeneration of Pd(0) for continuation of the catalytic cycle. If one assumes that all three steps are microreversible, the overall process can be considered to be driven thermodynamically, in most cases by the formation of MX. The catalytic cycle is applicable toward a wide range of Pd-catalyzed cross-coupling reactions between R^1^M and various organic halides R^2^X (R = allyl, propargyl, benzyl, acyl, alkenyl, alkynyl, aryl; listed in approximate order of reactivity).

**Figure 1.**
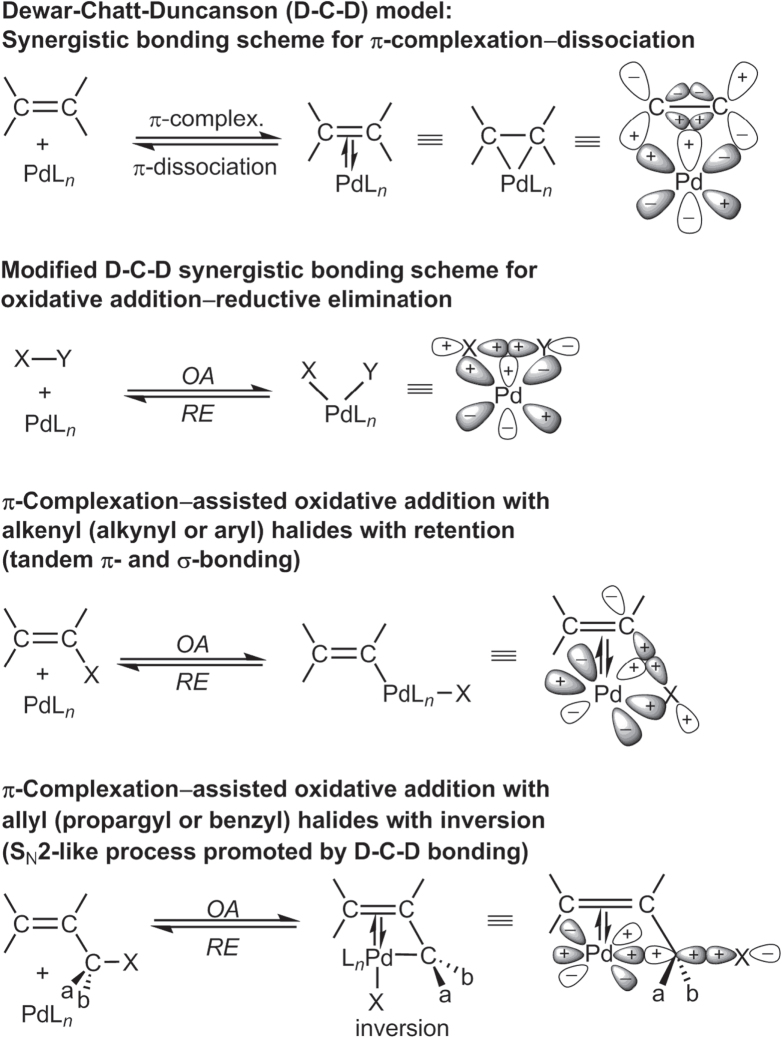
Reaction models based on frontier molecular orbital theory.

The catalytic cycle in scheme 1 serves as a reasonable model for other cross-coupling reactions mediated by Pd, Ni [25–27], and other TMs. Nevertheless, one must be mindful that few mechanistic schemes have been fully established for TM-catalyzed cross couplings. In most cases, these models should be considered as drawing boards for working hypotheses based on electron count or oxidation states. Some steps have also been described using molecular orbital theory, a more sophisticated approach to understanding TM-catalyzed cross-coupling. Reaction models have been proposed using concepts in frontier molecular orbital theory developed by Fukui [29], the conservation of orbital symmetry described by Woodward and Hoffman [30, 31], and synergistic bonding described by Dewar [32–34] as exemplified by the so-called Dewar–Chatt–Duncanson model (figure 1) [15]. These models suggest at least two critical factors in C–C cross-couplings catalyzed by *d*-block transition metals: (i) the availability of (filled) non-bonding orbitals on the organometallic species and one or more (unfilled) valence orbitals on the acceptor to serve as highest occupied and lowest unoccupied molecular orbitals respectively, and (ii) accessible redox states to support oxidative and reductive processes in the same reaction vessel.

**Scheme 2.**
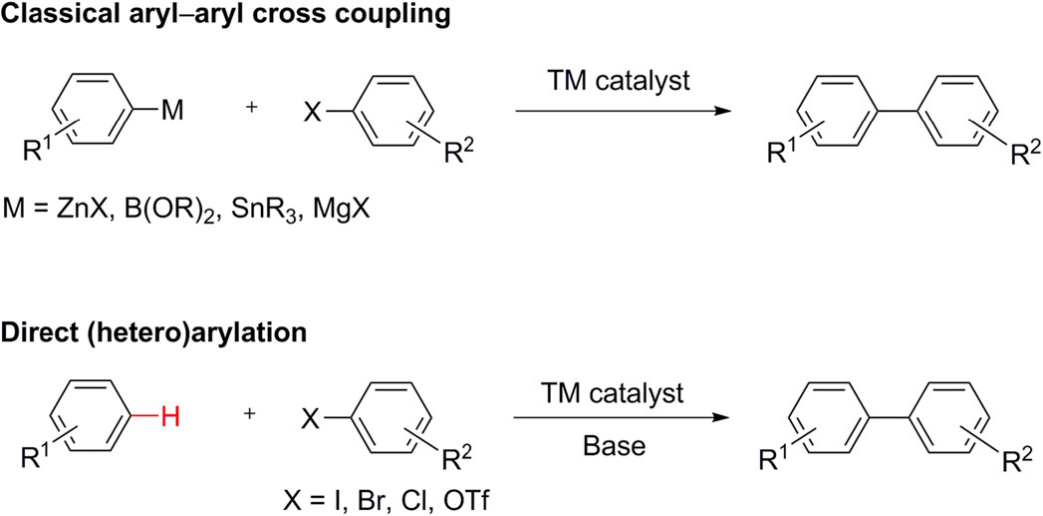
Classical aryl–aryl cross coupling versus direct (hetero)arylation.

In addition to these well-established classes of TM-catalyzed cross-coupling, arylation by direct C–H activation has recently emerged as a new type of cross-coupling reaction, and is being increasingly applied toward the synthesis of small molecules [35–37]. In principle, TM-catalyzed C–H activation does not require organic halides or the generation of organometallic intermediates in contrast to classical cross-coupling reactions (scheme 2), and is appealing from both environmental and economical perspectives. However, C–H activation can only be considered as an alternative in specific instances, due to two major hurdles: (i) most C–H bonds are kinetically inert and often require the assistance of a nearby directing group, which limits the substrate scope; (ii) organic molecules typically contain multiple C–H bonds, which increases the challenge of regiospecific activation. In this regard, it should be noted that an organometallic species can strongly influence the regiospecificity of C−H bond activation, prior to coupling. Therefore, organometallic intermediates will likely continue to serve as mainstays in TM-mediated cross-coupling reactions.

## 3. Synthesis of organic electronic materials

### 3.1. Polythiophenes and related structures

**Figure 2.**
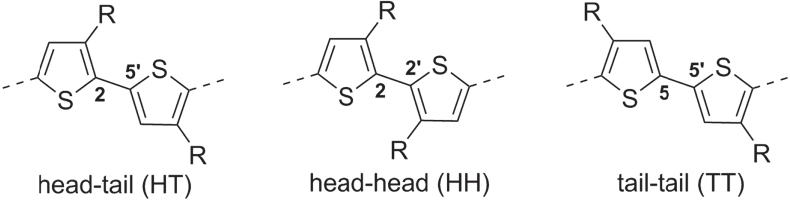
Three regioisomeric couplings of 3-substituted thiophenes.

Thiophene-based polymers and oligomers represent one of the largest classes of *π*-conjugated organic materials. Polythiophenes have low band gaps and are valued for their high charge carrier mobility at low voltage bias, and also for their excellent thermal and chemical stability [6]. Unsubstituted 2,5-polythiophenes are poorly soluble and difficult to work with, but their processability is greatly improved by introducing pendant groups at the C3 position. The intrinsic asymmetry of 3-substituted thiophenes gives rise to three types of coupling products: 2 → 5′ or head–tail (HT), 2 → 2′ or head–head (HH), and 5 → 5′ or tail–tail (TT) (figure 2). The steric interactions between C3 groups in HH-coupled units promote twisted, non-planar backbone conformations, leading to loss of *π*-conjugation and carrier mobility. For this reason, synthetic methods have been largely focused on the preparation of regioregular poly(3-alkylthiophene)s (P3ATs) with continuous HT couplings.

**Scheme 3.**
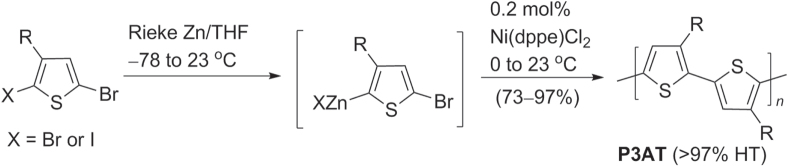
Regioregular synthesis of head-to-tail poly(3-alkylthiophenes) (HT-P3ATs) using Ni-catalyzed Negishi conditions: dppe = 1,2-bis(diphenylphosphino)ethane.

Regioregular P3ATs were first synthesized over 30 years ago by McCullough and co-workers [38], and further improved by the use highly reactive (Rieke) Zn to prepare regioregular P3ATs (scheme 3) [39, 40]. The zerovalent Zn undergoes oxidative addition with 2,5-dibromo-3-alkylthiophene or 2-bromo-5-iodo-3-alkylthiophene to afford an organozinc intermediate with > 98% regioselectivity, which can polymerize into HT P3ATs by Ni-catalyzed Negishi coupling. The regioregularity could be optimized to > 97% HT by controlling the reaction temperature and catalyst loadings. The molecular weights of P3ATs prepared by this method were about 30 kDa with a polydispersity index (PDI) near 1.4.

**Scheme 4.**
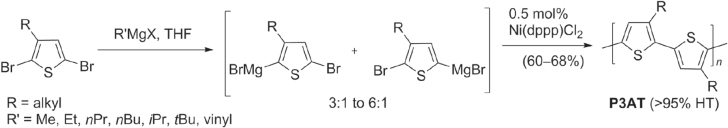
Regioregular HT-P3ATs prepared using Ni-catalyzed Kumada conditions: dppp = 1,2-bis(diphenylphosphino)propane.

P3ATs can also be synthesized using a Grignard metathesis (GRIM) protocol and Ni-catalyzed (Kumada-type) coupling [41, 42]. 2,5-Dibromo-3-alkylthiophene reacted with one equivalent of alkyl or vinyl Grignard reagent to form a mixture of intermediates by metal−halogen exchange, referred to as Grignard metathesis (scheme 4). Despite the apparent lack of regioselectivity during Grignard metathesis, Ni-catalyzed polymerization affords P3ATs with high regiospecificity (>95% HT) and low PDI (1.2–1.4), and molecular weights in the range of 20–35 kDa. In this case, the high regioselectivity was attributed to a combination of steric and electronic effects during the catalytic cycle [39].

**Scheme 5.**
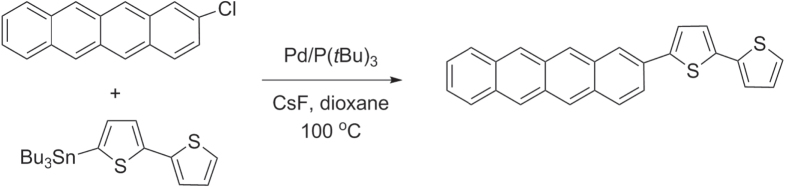
Tetracenyl bithiophene prepared using modified Stille conditions.

**Scheme 6.**
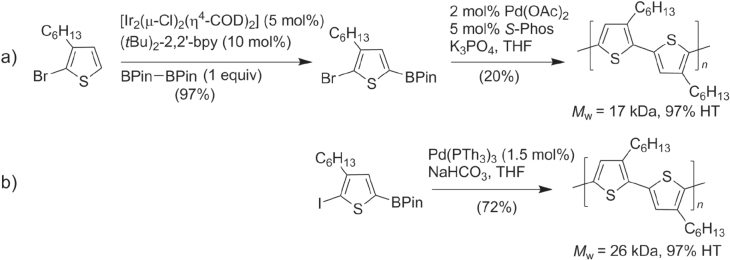
P3AT synthesis using modified Suzuki conditions (bpy = bipyridine; COD = 1,5-cyclooctadiene; Pin = pinacolato; *S*-Phos = P(cyclohexyl)_2_(2,6-(MeO)_2_biphenyl); Th = 2-thienyl).

In addition to the Negishi- and Kumada-type reactions, regioregular P3ATs have also been synthesized by other Pd-catalyzed cross-coupling reactions using organotins (Stille coupling) [43] and organoborons (Suzuki coupling) [44]. The ease of isolating and purifying these precursors has enabled numerous thiophene-based compounds to be synthesized from building blocks with widely different characteristics. However, application of Stille or Suzuki coupling toward polythiophene synthesis tends to produce P3ATs of lower molecular weight and/or regioregularity compared with Negishi- and Kumada-type conditions. This is gradually being improved by the development of new coordinative ligands; the use of sterically demanding phosphines [45] or *N*-heterocyclic carbenes [46, 47] has increased the scope of these couplings to include less activated substrates (e.g., aryl chlorides) in Pd-catalyzed cross-coupling reactions, while maintaining mild reaction conditions. For example, 2-chlorotetracene was efficiently coupled with a 5′-stannylbithiophene via a modified Stille coupling by using the electron-rich and sterically demanding ligand P(*t*Bu)_3_, to produce 5′-tetracenyl bithiophene for vapor deposition in organic TFT production (scheme 5) [48, 49]. Ligand-modified couplings can also increase the molecular weight and regioregularity of P3AT [50]. In the example below, P3AT synthesis with 5-bromothiophene and the ligand *S*-Phos produced P3AT under Suzuki-type conditions in low yields, but changing the monomer and ligand to 5-iodothiophene and P(2-thienyl)_3_ afforded P3AT in good yields, high molecular weight (*M*_w_ = 26 kDa), and 97% HT regioregularity (scheme 6) [51].

**Scheme 7.**
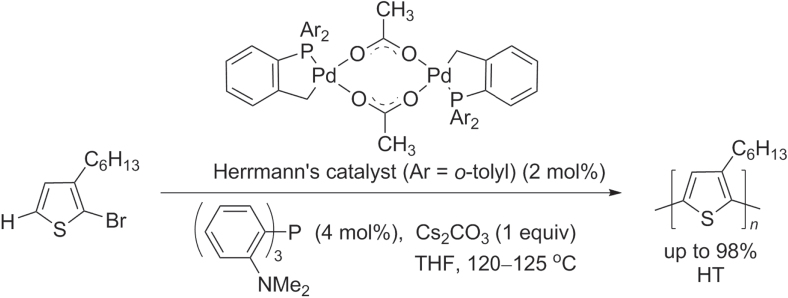
P3AT synthesis using Hermann’s catalyst for C−H bond activation.

The synthesis of regioregular P3ATs by direct C–H bond activation was first reported in 1999, using 2-halo-3-alkylthiophenes as monomers and Pd(OAc)_2_ as the catalyst [52]. Although the molecular weights of these polymers were initially low (*M*_w_ ∼ 3 kDa) and with 90% HT regioregularity, the Pd-mediated dehydrohalogenative polymerization of 2-bromo-3-hexylthiophene yielded regioregular P3ATs of higher molecular weight (*M*_w_ ∼ 30 kDa) when using the thermally stable bis-palladacycle known as Herrmann’s catalyst [53]. The catalytic efficiency could be further improved by adding tris(2-dimethylaminophenyl)phosphine as a stabilizing ligand, enabling the polymerization of 3-hexylthiophene into P3HT in 99% yield with high molecular weight (*M*_w_ > 30 kDa) and > 98% HT regioregularity (scheme 7).

**Figure 3.**
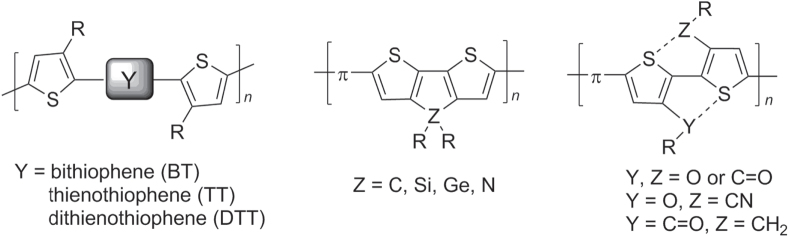
Polythiophene-like derivatives with low torsional strain between *π*-conjugated units.

Deviations from regioregularity in P3AT synthesis increases the number of head-to-head (HH) isomers, which adopt nonplanar conformations in order to reduce torsional strain. Several strategies have been developed to synthesize P3AT-like polymers with extended planar backbones whose conformations do not depend on regioregularity (figure 3). These include: (i) the incorporation of unsubstituted oligothiophenes as spacers within the P3AT chain, which effectively removes the steric interactions between 3AT units; (ii) locking the polymer backbones into planar conformations by using fused thiophenes, and (iii) introducing electronic stabilizing interactions between the ring sulfur and substituents of neighboring 3ATs to promote planarity between HH subunits [54]. Many of the modified P3ATs in figure 3 were synthesized by Pd-catalyzed cross-coupling using Stille conditions [47, 55, 56].

**Scheme 8.**
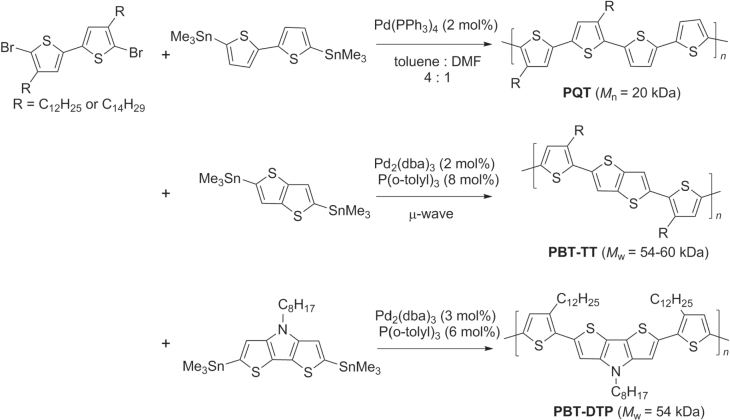
Synthesis of conductive polythiophenes with TT bithiophene subunits (PQT, PBT−TT, PBT−DTP) using Stille copolymerization: dba = dibenzylideneacetone; DMF = dimethylformamide.

Stille copolymerization has also been used to make P3HT-like polymers containing tail-to-tail bithiophene (TT−BT) units, such as poly(quaterthiophene) (PQT), poly(bithiophene−thieno(3,2-b]thiophene) (PBT−TT), and poly(bithiophene−dithieno(3,2-*b*:2′,3′-*d*)pyrrole) (PBT−DTP) (scheme 8). PQTs crystallize more readily than P3HTs, which induces greater long-range order between chains and subsequently higher hole mobilities (0.2–0.6 cm^2^ V^−1^ s^−1^) [57]. The TT−BT units in PBT−TT and PBT−DTP likewise promote polymer self-organization by minimizing steric interactions between neighboring alkyl groups and reinforcing backbone planarity, to produce crystalline domains on the order of 0.2 *μ*m (the resolution of printable channel lengths) and hole mobilities similar to that of PQTs [58, 59]. All of these *π*-conjugated polythiophenes have great promise as high-performance organic semiconductors.

**Scheme 9.**
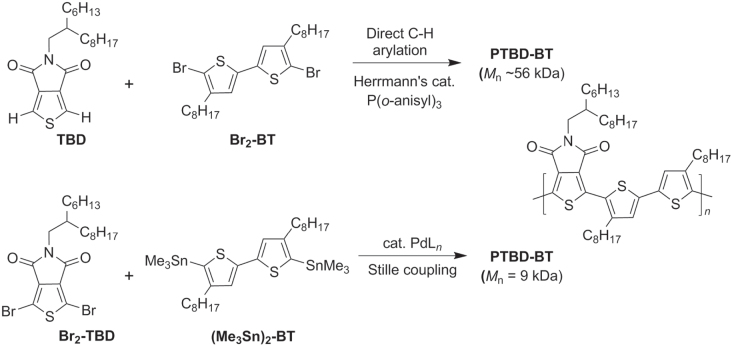
Comparison of Pd-catalyzed polymerization with direct C−H bond activation versus Stille coupling in the synthesis of PTPD-BT.

Direct C−H activation can also be used to produce *π*-conjugated polymers with TT−BT units, as exemplified in the polycondensation of thieno(3,4-c)pyrrole-4,6-dione (TPD) with a 3,3′-dioctyl-BT derivative (scheme 9) [60]. Poly(TPD-BT) has excellent performance characteristics in both solar cells and field-effect transistors, and the imide within the TPD unit may serve as a directing group in the C–H bond activation of TPD. Polymerization by direct C–H heteroarylation was performed using Herrmann’s catalyst with P(*o*-anisyl)_3_, which produced PTPD-BT in 96% yield with high molecular weight (*M*_w_ = 56 kDa). In comparison, Pd-mediated cross-coupling with dibromo-TPD and distannyl-BT using Stille coupling conditions gave PTPD-BT in 71% yield with much lower molecular weight (*M*_w_ = 9 kDa).

**Scheme 10.**
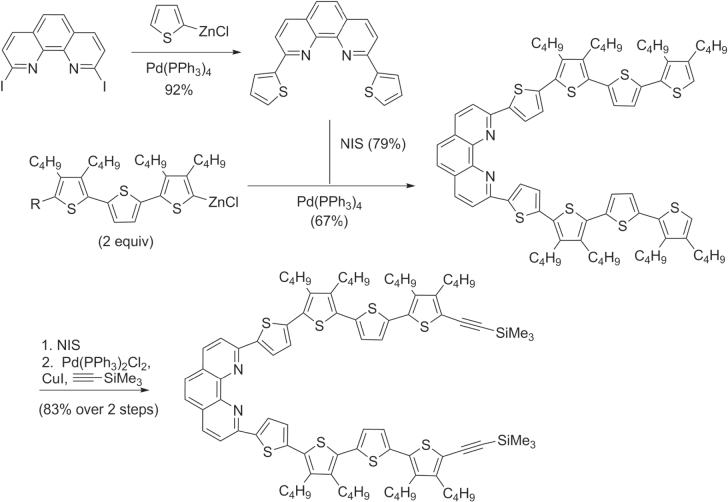
Phenanthroline-bridged oligothiophenes prepared by Negishi cross-coupling. NIS = *N*-iodosuccinimide.

With regard to oligothiophenes and related molecules, Pd-catalyzed Negishi cross-coupling reactions have been used to prepare a variety of *π*-conjugated molecules using halogenated heterocycles and zincated oligothiophenes. Novel polyheteroaromatic systems containing two oligothiophene subunits bridged by phenanthroline derivatives have been prepared as metal-chelating units for electronically-based chemical sensors [61], and as intermediates for *π*-conjugated catenanes and other topologically interesting structures (scheme 10) [62].

**Scheme 11.**
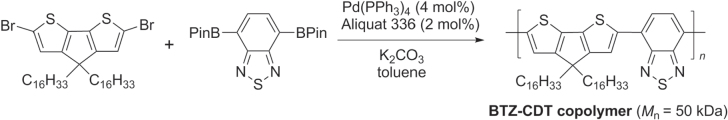
Benzothiadiazole−cyclopentadithiophene (BTZ-CDT) copolymers with exceptionally high hole mobilities, prepared by Suzuki polycondensation. Aliquat 336 = trioctyl-methylammonium chloride; Pin = pinacolato.

A high molecular-weight copolymer of benzothiadiazole (BTZ) and cyclopentadithiophene (CDT) has been prepared by Suzuki polycondensation (*M*_n_ = 50 kDa, scheme 11) [63]. This *π*-conjugated polymer has a strong propensity to self-assemble into lamellar stacks, supporting hole mobilities as high as 1.4 cm^2^ V^−1^ s^−1^. Further optimization produced BTZ-CDT copolymers of even greater crystallinity with *I*_on_/*I*_off_ ratios of over 10^5^ and hole mobilities of 3.3 cm^2^ V^−1^ s^−1^, among the highest values reported so far for polymer-based TFTs [64].

### 3.2. Polyfluorenes and related structures

Polyfluorenes (PFs) are conformationally constrained polyarylenes that are comprised of *π*-conjugated biphenyl units bridged by *sp* ^*3*^ carbon atoms (C9 position). PFs are well known for their strong electroluminescence with emissions at blue wavelengths [65]. A variety of aryl and alkyl substituents have been introduced at C9 to solubilize these rigid polymers; these can also modulate the electronic or photophysical properties by applying torsional strain to the *π*-conjugated backbone. The electrochromic properties of PFs have made them one of most widely used organic electronic materials, with applications in organic light-emitting diods (OLEDs), lasers, TFTs, and sensors [66]. These tunable materials have also given rise to *π*-conjugated polymers containing spirofluorene units and related polycyclic derivatives, many of which have been described in earlier reviews [67, 68].

**Scheme 12.**
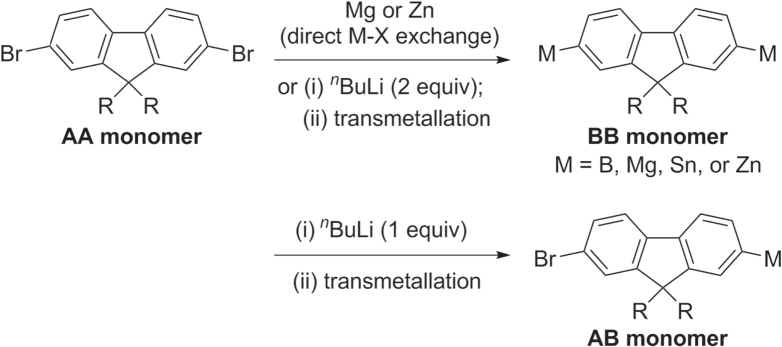
Preparation of monomers for PF synthesis.

Oligo- and polyfluorenes are often synthesized using TM-catalyzed polycondensation, which can be sorted into three categories: (i) homopolymerization of 2,7-dibromofluorenes (AA monomer) via Ni-catalyzed Yamamoto coupling; (ii) copolymerization of AA monomers with their bismetallated congeners (BB monomers) via Pd-catalyzed cross-coupling (Kumada, Negishi, Stille, or Suzuki conditions); and (iii) homopolymerization of monometallated bromofluorenes (AB monomers) via Pd-catalyzed cross-coupling. AB and BB-type monomers are readily prepared from 2,7-dihalofluorenes (AA-type monomers) by metal−halogen exchange using one or two equivalents of *n*-butyllithium or Grignard reagent, followed by transmetallation with borates, ZnBr_2_ or Me_3_SnCl (scheme 12) [69–72].

**Scheme 13.**
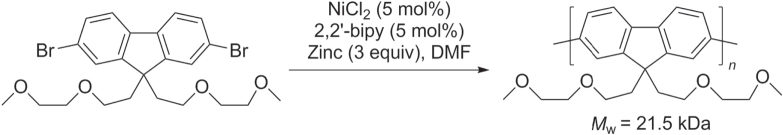
Homopolymerization of AA-type monomer into a poly(9,9-bis(3,6-dioxaheptyl)-fluorene-2,7-diyl) by Ni-catalyzed Yamamoto coupling. bipy = 2,2′-bipyridine; DMF = dimethylformamide.

Yamamoto polymerization of a dihaloaromatic compound (AA-type monomer) gives a polymer. The main advantage of Yamamoto polymerization is that AA-type monomers are straightforward to work with. For example, poly(9,9-bis(2-ethylhexyl)fluorene) was prepared by Yamamoto-type polycondensation of a 2,7-dibromofluorene with bis(COD)nickel and bipyridine [73]. However, this method usually requires stoichiometric amounts of catalyst. Investigations on Ni-catalyzed reductive polymerization of 2,7-dibromofluorenes with excess zinc dust as a reductant led to the synthesis of highly conjugated and processable PFs (scheme 13) [74].

**Scheme 14.**
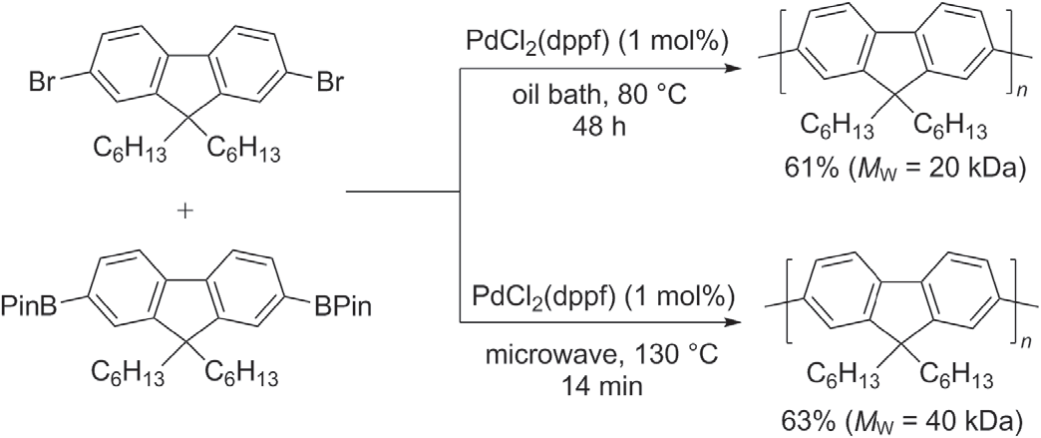
Heteropolymerization of AA- and BB-type monomer into poly(9,9-dihexyl)-fluorene by Suzuki condensation under conventional or microwave-assisted heating conditions. dppf = 1,1′-bis(diphenylphosphino)ferrocene; Pin = pinacolato.

Pd-catalyzed heteropolymerization of AA- and BB-type monomers and homopolymerization of AB-type monomers are all used to prepare polyfluorenes. Suzuki conditions have been found to be efficient and have become widely adopted for the synthesis of high molecular weight PFs (50−60 kDa) [75, 76]. Suzuki couplings can be accelerated by over two orders of magnitude by using microwave heating, with increased chain propagation: heteropolymerization of 2,7-dibromo- and 2,7-di(pinacolato)boryl)-9,9-dihexylfluorene under microwave conditions produced PFs in under 15 min at twice the molecular weight (*M*_w_ = 40 kDa), compared with that synthesized by conventional heating for 48 h (*M*_w_ = 20 kDa) (scheme 14) [77].

**Scheme 15.**
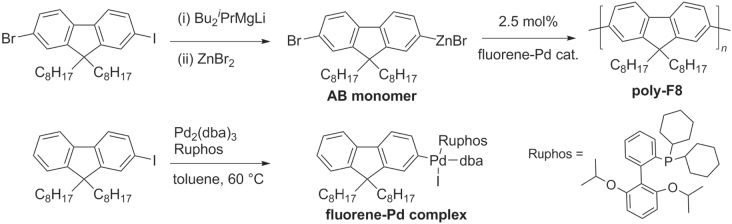
Homopolymerization using Negishi coupling conditions with an air-stable fluorene−Pd complex: dba = dibenzylideneacetone; Ruphos = 2′,6′-bis(1-methylethoxy)(1,1′-biphenyl)-2-yl)dicyclohexylphosphine.

Chain-growth polycondensations of AB-type monomers are a popular alternative for the preparation of well-defined *π*-conjugated polymers [78]. Propagation typically involves an intramolecular catalyst-transfer pathway, following formation of a TM-based initiator. Recently there has been much progress in chain-growth polycondensation using TM complexes with bulky and electron-rich ligands. For example, a universal chain-growth polymerization initiated with aryl-Pd(Ruphos)-X species has been developed using a Negishi coupling protocol, which is applicable to the synthesis of PFs, P3HT, and other *π*-conjugated copolymers [72]. 2-Bromo-7-iodo-9,9-dioctylfluorene (F8) was converted into an AB-type organozinc monomer, then polymerized at room temperature into poly-F8 using a fluorene−Pd complex as an initiator (scheme 15). It is worth noting that the fluorene−Pd initiator (prepared from the corresponding 2-iodofluorene) is air-stable and does not require anhydrous conditions, and therefore is very easy to handle.

**Scheme 16.**
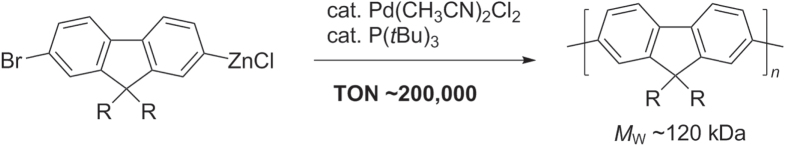
Pd/P*t*Bu_3_-catalyzed Negishi chain-growth polycondensation of AB-type monomers proceeds with exceptionally high catalytic efficiency.

Pd/P*t*Bu_3_-catalyzed Negishi chain-growth polycondensation have recently been reported to produce polyfluorenes with molecular weights of up to 120 kDa (scheme 16), with exceptionally high catalyst turnover numbers (TON > 200 000, the highest reported to date for TM-catalyzed cross-coupling polycondensations) and turnover frequencies (TOFs up to 280 s^−1^) [79]. These remarkable catalytic efficiencies can result in TONs and TOFs that are two orders of magnitude higher than that of step-growth polycondensation of functionally-related AA/BB-type monomers.

**Scheme 17.**
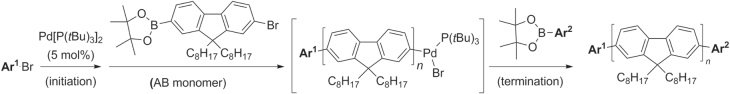
Heterobis-functionalized oligofluorenes by Pd-catalyzed Suzuki coupling.

The Pd-catalyzed oliogomerization of fluorene derivatives can also be controlled to the extent that discrete ‘molecular wires’ with different end groups can be designed and synthesized. Polymerization could be initiated from a bromoarene (Ar^1^-Br) by oxidative addition of Pd(P*t*Bu_3_)_2_ to generate a Pd(P*t*Bu_3_)(Ar)Br complex, similar to that featured above, followed by chain growth using Suzuki coupling conditions with AB-type monomer then terminated by addition of a arylborate ester (Ar^2^-B(OR)_2_) to produce oligofluorenes (*n* = 10–23) in high yields with full control over the end groups (scheme 17) [80]. The propagation conditions can be tuned to produce polyfluorenes in fair yields with polydispersities of 1.2–1.4, with *M*_*n*_ values in the range of 5–10 kDa (12–23 subunits).

**Scheme 18.**
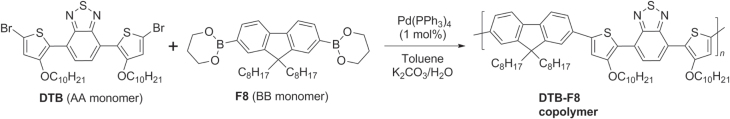
Hybrid copolymer with alternating DTB and fluorene units, prepared by Suzuki polycondensation.

Activated fluorenes can be copolymerized with other *π*-conjugated systems to create hybrid PFs, allowing further tuning of the electronic band structures and emission wavelengths. This is generally achieved by the Pd-catalyzed cross-coupling of alternating AA- and BB-type monomers, using either component as the bismetallated species. For example, a hybrid copolymer comprised of alternating 4,7-bis-(3-decyloxythiophen-2-yl)benzothiadiazole (DTB) and 9,9-dioctylfluorene (F8) was synthesized by Suzuki polycondensation (scheme 18) [81]. The alternating copolymer is regioregular by design, and the alkoxy substituents on the thiophene rings effectively reduce the band gap for enhanced charge transfer to nearby electron acceptors (*n*-type materials). These attributes make DTB-F8 copolymer an attractive candidate for improving electron and hole mobility in polymer-based solar cells.

**Scheme 19.**
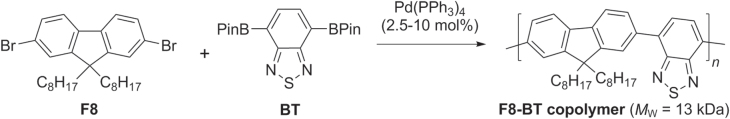
Electroluminescent dioctylfluorene−benzothiadiazole (F8−BT) copolymer, prepared by dispersion polymerization using Suzuki conditions.

Copolymers of alternating 9,9-dioctylfluorene (F8) and benzothiadiazole (BT) units have been prepared by dispersion polymerization using Suzuki conditions (scheme 19) [82]. F8−BT copolymers exhibit highly dispersive electron transport properties, with electron mobilities of the order of 10^−3^ cm^2^ V^−1^ s^−1^; it also produces efficient luminescence near 550 nm and is thus a promising candidate for OLEDs [83]. Dispersion polymerization also produces uniform particles that can self-assemble into photonic crystals with well-defined bandgaps, and can be applied toward the preparation of monodisperse particles made from other PF-like polymers [83].

**Scheme 20.**
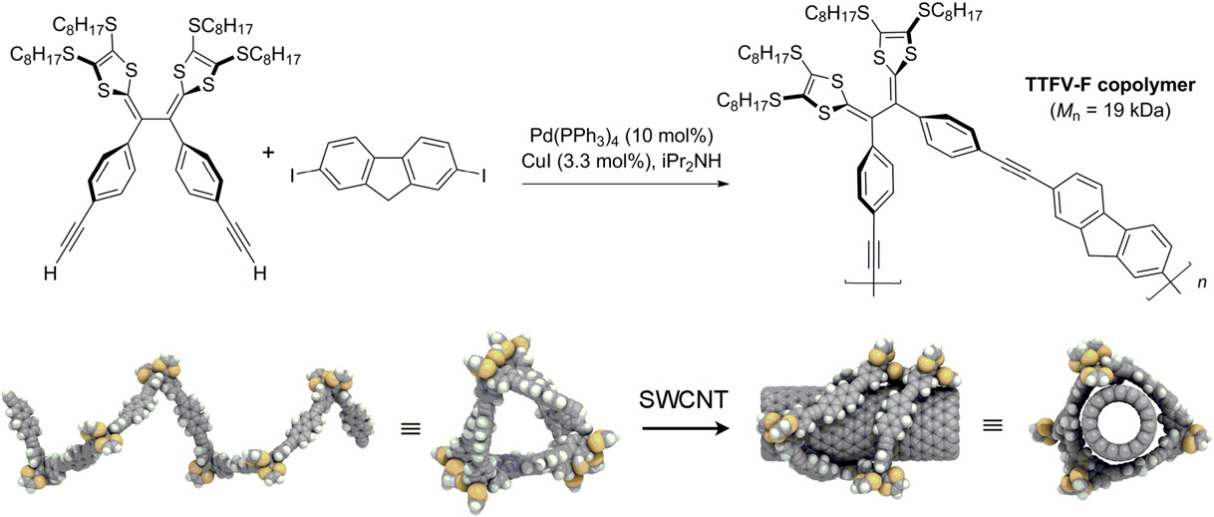
Top, synthesis of helical tetrathiafulvalene−fluorene (TTFV-F) copolymers by Sonogashira coupling. Bottom, helical conformation of TTFV-F, before and after complexation with SWCNT [84]. Reprinted with permission from the American Chemical Society.

Tetrathiafulvalene−fluorene (TTFV-F) copolymers with protohelical conformations, which have been prepared by Sonogashira polycondensation, have been shown to undergo large conformational changes upon protonation (scheme 20) [84]. TTFV-F copolymers were capable of forming stable dispersions of single-walled carbon nanotubes (SWCNTs) in toluene under neutral conditions, but complexation was reversed upon addition of acid.

**Scheme 21.**
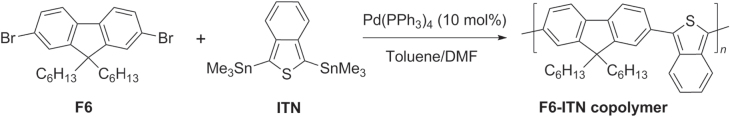
Copolymer of alternating F6 and ITN units, prepared by Stille polycondensation.

Fluorene derivatives have also been copolymerized with isothianaphthene (ITN), a bicyclic compound with attractive electric and optical properties for generating low-bandgap polymers based on its *o*-quinoidal character [85]. Copolymers with alternating 9,9-dihexylfluorene (F6) and ITN units have been synthesized by Stille polycondensation (scheme 21) [86]. Bulk heterojunction photovoltaic (PV) devices constructed using a blend of F6−ITN copolymer and a fullerene derivative gave power conversion efficiencies ranging from 0.12% to 0.43%. Many other examples of fluorene-based conjugated polymers exist, and have been nicely summarized in another review [67].

**Scheme 22.**
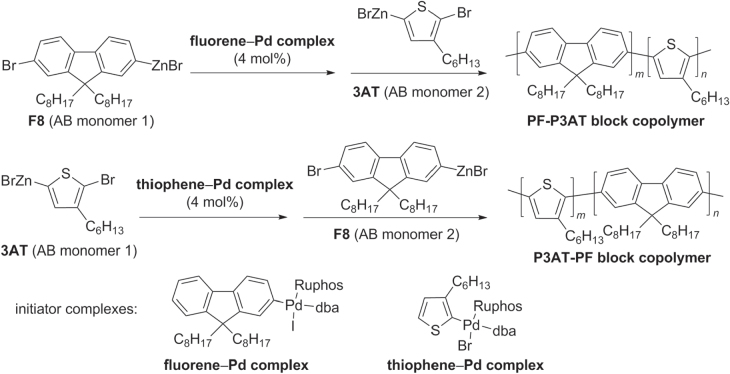
Synthesis of diblock *π*-conjugated PF−P3AT copolymers based on a Negishi coupling protocol, by sequential addition of AB-type monomers: dba = dibenzylideneacetone; Ruphos = 2′,6′-bis(1-methylethoxy)(1,1′-biphenyl)-2-yl)dicyclohexylphosphine.

Block copolymers with extended *π*-conjugation have also been synthesized, using Negishi-type couplings for the sequential polymerization of AB-type monomers with Pd complexes as polymer initiators [72]. Copolymers comprised of F8 and 3AT segments have been prepared by the stepwise polymerization of the corresponding AB-type monomers, catalyzed either by fluorene−Pd complex (to initiate growth of PF block) or thiophene−Pd complex (to initiate growth of P3AT block) (scheme 22). The modularity of this block copolymer synthesis is remarkable, and is a significant advance over previous protocols involving the block copolymerization of *π*-conjugated units having different electronic characteristics [87, 88].

**Scheme 23.**
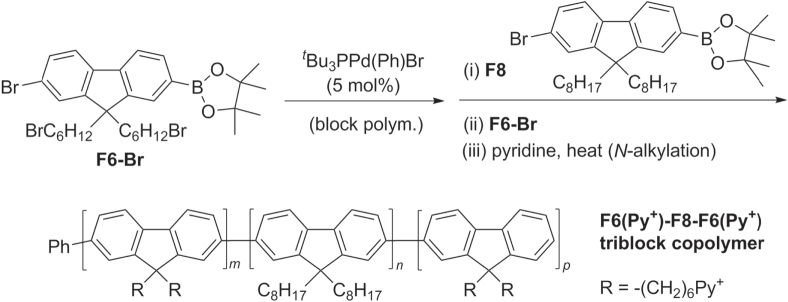
PF-based triblock copolymer via Suzuki polymerization. Py^+^ = pyridinium.

Triblock *π*-conjugated copolymers have also been synthesized by Pd-catalyzed cross-coupling using Suzuki-type protocols, with narrow molecular weight distributions. PF-based copolymers were prepared using AB-type monomers derived from di(6′-bromohexyl)fluorene (F6-Br) and dioctylfluorene (F8) using (^*t*^Bu_3_P)Pd(Ph)Br as the catalyst [89]. The terminal bromides on F6-Br could be substituted with pyridine (F6-Py^+^) to introduce polyelectrolyte character at both ends of the triblock copolymer (scheme 23).

**Scheme 24.**
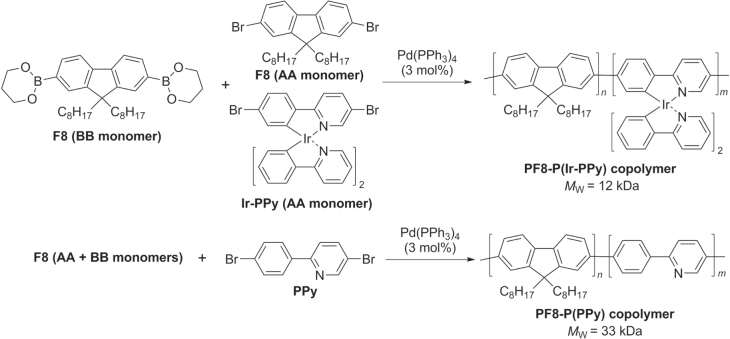
Polycondensation of Ir-containing PF derivatives using Suzuki conditions.

Photovoltaic properties such as charge generation, power conversion efficiency (PCE), and external quantum efficiency (EQE) can be enhanced in *π*-conjugated polymers by the inclusion of select transition metals. Such an effect has been investigated by the incorporation of Ir into a PF-based backbone, which was prepared by Suzuki condensation of BB monomer (diboryl-F8) with AA-type-monomers generated from F8 and Ir-substituted phenylpyridine (Ir-PPy) (scheme 24) [90]. A *π*-conjugated copolymer PF8-P(PPy) without Ir was prepared as a control. Ir-containing copolymer PF8-P(Ir-PPy) produced a maximum EQE at 350 nm of 10.3% and a PCE of 0.07%, more than an order of magnitude larger than those measured from its non-Ir counterpart PF8-P(PPy) (1.1% and 0.002% respectively). The enhanced performance of PF8-P(Ir-PPy) has been attributed to the formation of long-lived triplet excitons with extended diffusion lengths.

### 3.3. Polyphenylenes and poly(heteroarylene)s

Poly- and oligoarylenes have been developed as basic model compounds for studying redox properties and understanding of the spectroscopic data [91]. These compounds are frequently synthesized by the Pd-catalyzed polycondensation of AA and BB monomers. The exceptional physical properties of polyphenylenes and related compounds have supported the development of organic electronic materials that are highly processable [80, 92, 93].

**Scheme 25.**
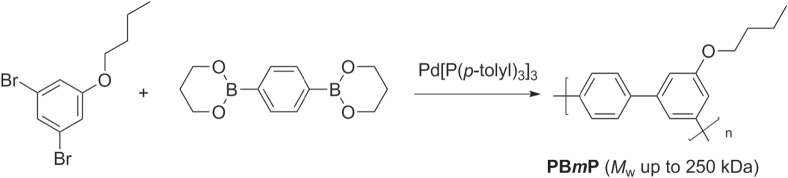
Synthesis of protohelical poly(3-butoxy-4′,5-*meta*-biphenylene (PB*m*P) by Suzuki polycondensation.

Suzuki polycondensation has been applied in the synthesis of poly(3-butoxy-4′,5-*meta*-biphenylene) (PB*m*P), a novel example of a protohelical *π*-conjugated polymer (scheme 25) [94]. Butyl 3,5-dibromophenyl ether was mixed with 1,4-phenylene diborate ester using standard Suzuki polycondensation conditions to yield PB*m*Ps with *M*_*w*_ values as high as 250 kDa after fractionation, and glass transition temperatures (*T*_*g*_) in the range of 150–166 °C. The high-*M*_*w*_ PBmP is remarkably tough, with yield stress and tensile strength comparable to that of conventional thermoplastics such as polycarbonate (PC) and poly(methyl)methacrylate (PMMA). Suzuki polycondensation can also provide ready access to monodisperse *meta*-substituted oligo- and polyphenylenes, which also have helical conformations and are popular structural motifs in the design of foldamers and shape-persistent organic polymers [95].

**Scheme 26.**
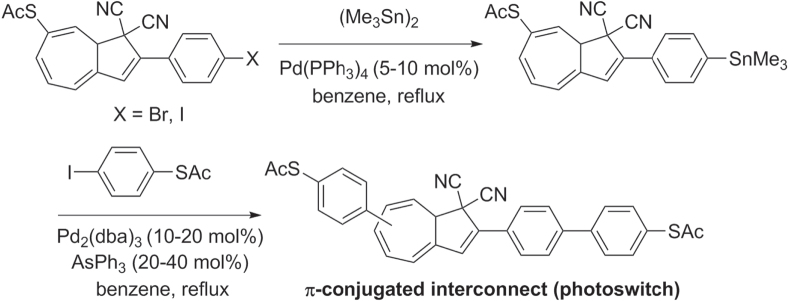
Functionalizations via Stille couplings: dba = dibenzylideneacetone.

Stille cross-coupling conditions have been used to synthesize poly- and oligoarylenes in the presence of sensitive functional groups, a limitation frequently encountered in the Suzuki reaction. For example, bis(thioacetyl)oligoarenes designed as potential molecular ‘alligator clips’ were prepared by using triphenylarsine as an ancillary ligand, without adverse effect on the thiol-containing units [96] (scheme 26). A short reaction time was necessary to minimize competitive side reactions involving the thioacetyl groups. The dihydroazulene core within this molecular interconnect has been proposed to function as a photoactive switch.

**Scheme 27.**
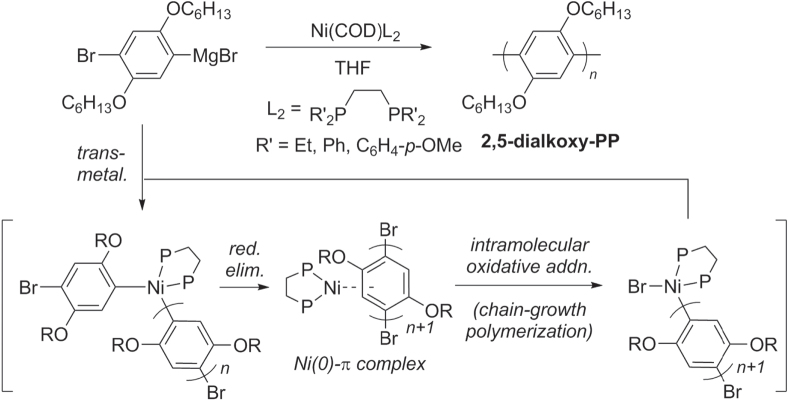
Improvement in chain-growth polymerizations based on Kumada cross-coupling, using electron-rich diphosphine ligands.

Kumada cross-couplings can also be used to make polyphenylenes, especially in the industrial-scale production of organic electronic materials. This reaction has been proposed to proceed through a Ni(0)−arene *π*-complex after reductive elimination, an intermediate which can participate either in intermolecular chain transfer through a dissociative mechanism, or in intramolecular oxidative addition to support chain-growth polymerization (scheme 27). The latter pathway may be optimized by using bidentate phosphine ligands to accelerate the oxidative addition step [97]. Electron-rich diphosphine ligands have been found to be particularly effective at reducing competing chain transfer processes, and have strong potential for further development of Kumada-type polymerization catalysts.

**Scheme 28.**
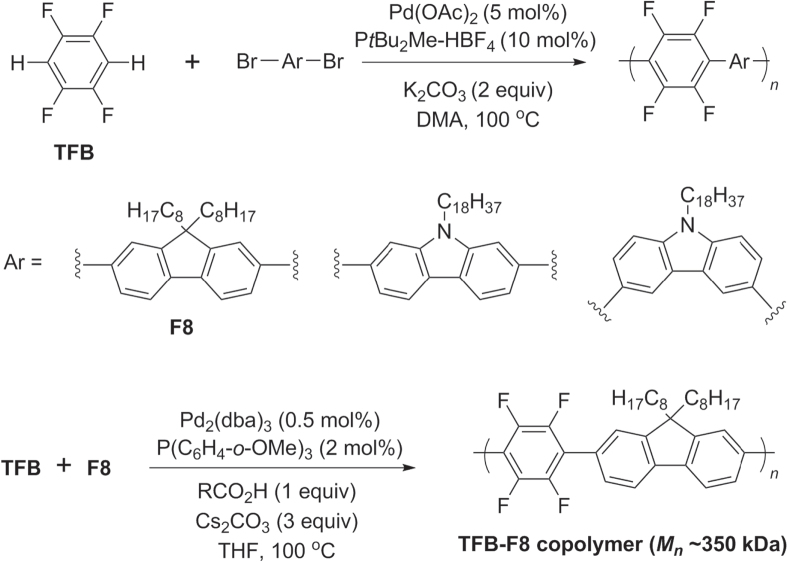
Pd-catalyzed C−H activation and polycondensation of tetrafluorobenzene (TFB) into heteroarylene copolymers. Ac = acetyl; dba = dibenzylideneacetone; DMA = dimethylacetamide.

Direct C−H bond activation has recently been used to prepare heteroarylene copolymers containing electron-deficient aromatic units. Copolymerization of 1,2,4,5-tetrafluorobenzene (TFB) with brominated dioctylfluorene (F8) or *N*-alkylcarbazoles has been achieved in the presence of Pd(OAc)_2_ with P*t*Bu_2_Me and base (scheme 28) [98, 99]. The regioselectivity in C−H bond activation can be rather sensitive to the solvent system and reaction time, a significant limitation in direct arylation protocols [93]. On the other hand, direct arylation conditions can produce *π*-conjugated polymers of exceptionally high molecular weight (*M*_*n*_ ∼ 350 kDa), as demonstrated by the copolymerization of TFB and dibromo-F8 [100].

**Scheme 29.**
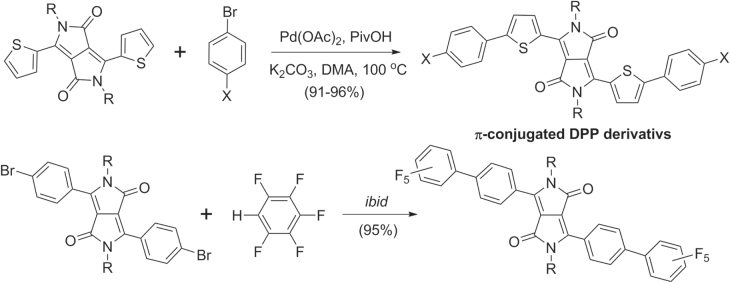
Direct C−H activation in the synthesis of *π*-conjugated diketopyrrolopyrroles (DPPs). DMA = dimethylacetamide; Piv = pivaloyl.

Pd-catalyzed direct arylation has also been used to prepare a variety of diketopyrrolopyrrole (DPP) derivatives [101]. Bisaryl-DPP heterotrimers were synthesized in excellent yields from dithienyl-DPP and bromoarenes, and also from dibromo-DPP and pentafluorobenzene (scheme 29). It is worth noting that Pd-mediated cross-coupling by C−H activation overcomes the hurdle of generating organometallic precursors from electron-deficient species such as perfluoroarenes. Furthermore, C−H activation in the absence of phosphine ligands can provide higher rates during cross coupling, relative to Suzuki or Stille conditions.

### 3.4. Oligo- and poly(arylethynylene)s

**Figure 4.**
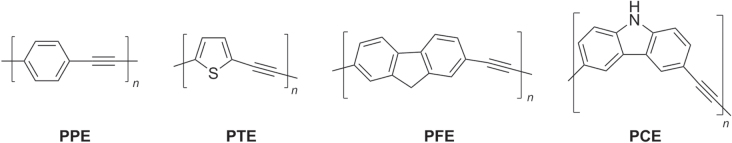
Structures of representative PAEs. PPE = poly(phenylene ethynylene), PTE = poly(thienylene ethynylene), PFE = poly(fluorene ethynylene), PCE = poly(carbazoyl ethynylene).

Over the last 25 years, poly(arylethynylene)s (PAEs) have received much attention as rigid, *π*-conjugated interconnects, with applications as polarizing materials in liquid crystal displays [102, 103], as molecular wires [104], and in the chemical detection of explosives and other volatile compounds [105, 106]. A wide variety of PAEs with different functional unit structures have been synthesized and studied [107], some examples of which are shown in figure 4. Despite their structural variety, they all share the same backbone feature, i.e. they are conjugated through ethyne-linked aromatic or heteroaromatic rings.

The most common approach to preparing PAEs is by Sonogashira coupling, a cross-coupling reaction between aromatic halides and terminal alkynes in the presence of catalytic CuI [24, 108]. This Pd-mediated coupling was in fact first described without Cu(I) by Heck [109] and Cassar [110], but required neat conditions at high temperatures or the use of strong base. In the Sonogashira−Hagihara reaction, the reaction is much milder and thus more compatible with various functional groups, and the addition of CuI as co-catalyst further enables the couplings to proceed at ambient temperatures.

**Scheme 30.**
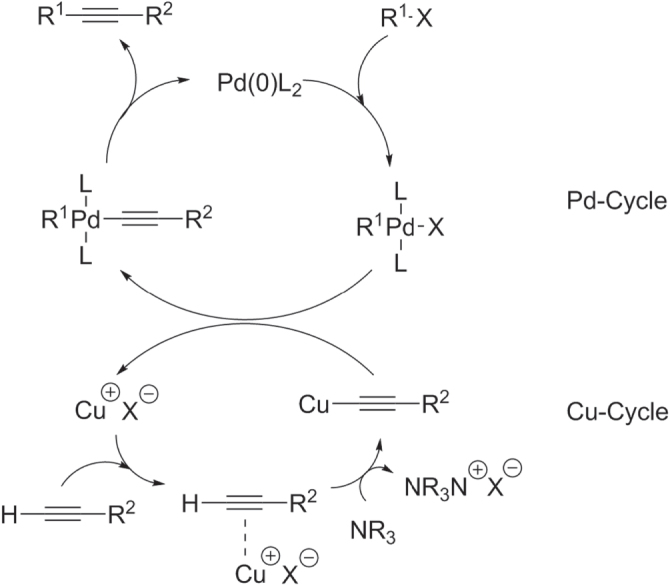
Working mechanism of the Pd/Cu-catalyzed Sonogashira reaction.

The mechanism of Sonogashira coupling has been a subject of considerable debate, but many agree that the proposed mechanism involves two independent catalytic cycles (scheme 30). In the Pd-catalyzed cycle, oxidative addition of electron-deficient Pd onto an aromatic halide (ArX) generates an aryl-PdX complex; in the Cu-catalyzed cycle, the alkyne, Cu(I) salt and base react to produce a Cu–acetylide intermediate. The two cycles then converge via transmetallation to generate an aryl-Pd–acetylide species, followed by reductive elimination to produce the alkyne with regeneration of electron-deficient Pd. It should be noted that many variants of the Sonogashira coupling also exist, including Cu-free variants and the use of Zn-acetylides.

**Scheme 31.**
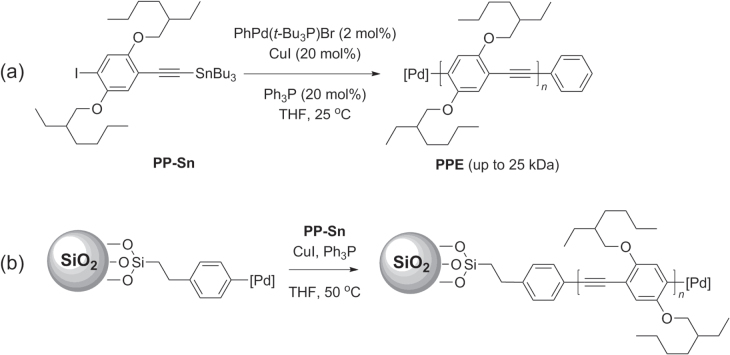
(a) Synthesis of PPEs with controlled polydispersity by catalytic transfer polycondensation (CTP) using Sonogashira-like conditions. (b) Surface-initiated polymerization of PPE under similar conditions.

While Sonogashira coupling has been successfully used to synthesize a variety of PAEs, there are also several shortcomings of this methodology, including the homocoupling of terminal alkynes, dehalogenation or substitution by phosphine ligands [111], and the relatively low degree of polymerization (DP < 100) which is strongly affected by the electronic structure of the monomers [112]. This has led to substantive efforts to improve Sonogashira coupling conditions to generate PPEs of either low polydispersity or high molecular weight. A catalyst transfer polycondensation (CTP) protocol using PhPd(*t*-Bu_3_P)Br has recently been developed as a ‘living’ chain growth process (as opposed to step growth) to afford poly(*p*-phenylethynylene)s (PPEs) of well-defined size and polydispersity (scheme 31) [113]. The use of a stannylated phenylethynyl monomer (PP-Sn) permitted a highly efficient conversion to PPEs with *M*_n_ values up to 25 kDa, and with chain lengths proportional to the initial monomer-to-catalyst ratio while maintaining a PDI below 1.4. A similar degree of control was observed when applying the CTP protocol to surface-initiated growth of PPE brushes on functionalized silica particles. The stoichiometric control of chain length offered by the CTP protocol has a number of important ramifications for the synthesis of *π*-conjugated polymers in general [42, 78].

**Scheme 32.**
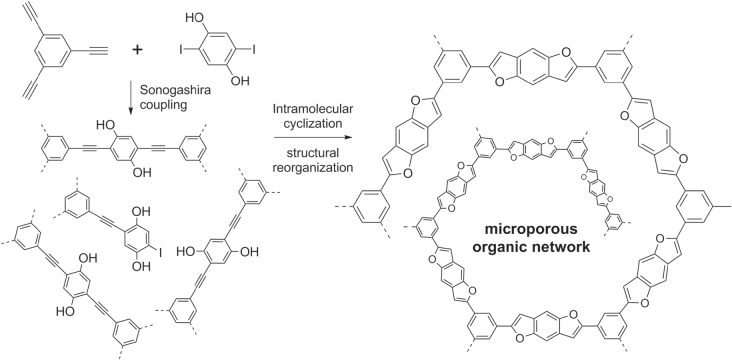
Synthesis of a benzodifuran-containing microporous organic network (MON) from a hyperbranched PPE precursor.

PPEs can also serve as precursors for novel hybrid copolymers, some having highly nonlinear conformations or branched structures [114–116]. For example, PPE-like networks were prepared by polycondensation of 1,3,5-tris(ethynyl)benzene and 2,5-diiodohydroquinone using Sonogashira conditions, then transformed into hyperbranched networks containing benzodifuran units formed by intramolecular cyclization, followed by macromolecular reorganization to produce a microporous organic network (scheme 32) [117].

**Scheme 33.**
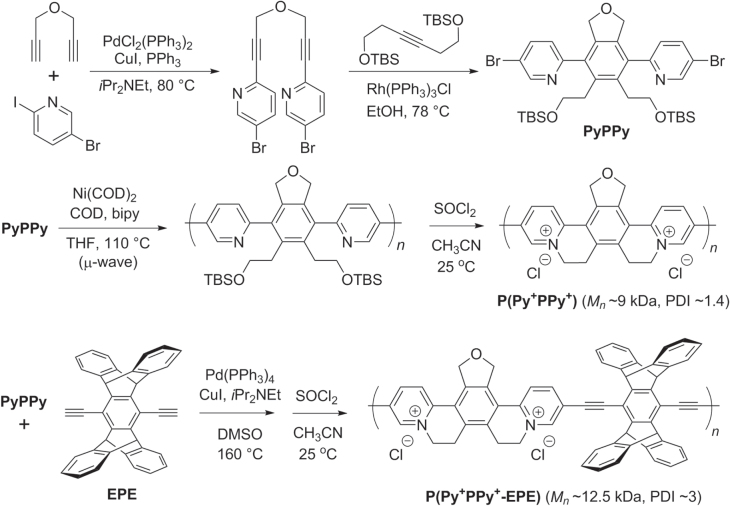
Synthesis of redox-active copolymers having rigid, *π*-conjugated backbones, using Yamamoto and Sonogashira coupling conditions. Bipy = 2,2′-bipyridine; COD = cyclooctadiene; EPE = ethynyl(pentiptycenyl)ethynylene; Py^+^PPy^+^ = pyridinium(phenyl)pyridinium; TBS = *t*-butyldimethylsilyl.

In another example featuring multiple Sonogashira couplings, substituted pyridines were coupled to dipropargyl ether then subjected to a Rh-catalyzed [2 + 2 + 2] cycloaddition to produce a 1,4-phenylene-bridged bipyridine (PyPPy, scheme 33) [118]. This intermediate could be polymerized by the Ni-catalyzed Yamamoto reaction under microwave conditions, followed by intramolecular alkylations to afford poly(pyridiniumphenylene) or P(Py^+^PPy^+^), an electronically active homopolymer with a rigid, *π*-conjugated backbone and high water solubility. Alternatively, PyPPy could be copolymerized with pentiptycene diacetylene under Sonogashira conditions, followed by pyridinium formation to produce a Py^+^PPy^+^−ethynylpentiptycenylethynylene (EPE) copolymer with hydrophobic pockets for guest inclusion. Both of these redox-active polymers display good charge-transport upon *n*-doping, and produce a strong optical response to electron-rich analytes with high electron affinity.

**Scheme 34.**
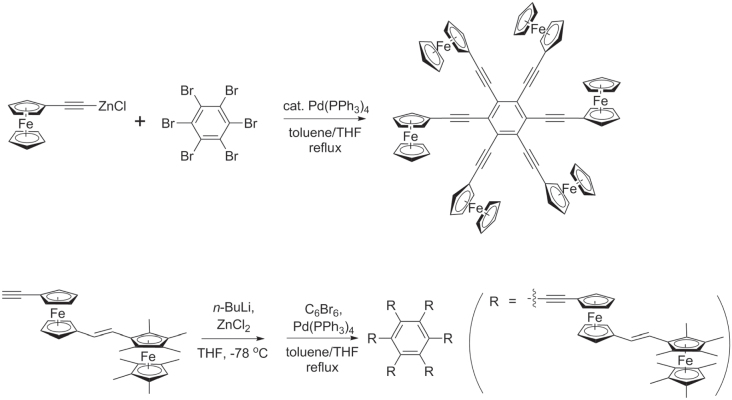
One-pot syntheses of hexakis(ferrocenylethynyl)benzene and hexakis((*E*)-(2-(ethynylferrocenyl)ethynyl)octamethylferrocene)benzene, using multiple Negishi couplings.

**Scheme 35.**
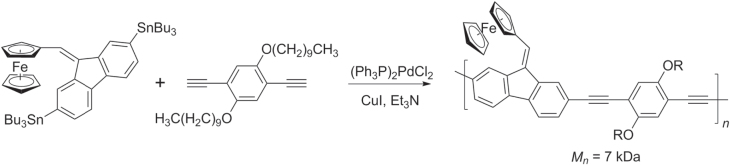
Synthesis of hybrid poly(aryl−fluorenylferrocenyl)ethynylenes by Stille coupling.

Negishi and Stille cross couplings have been used to synthesize redox-active, star-shaped oligo(phenylethynylene)s containing metal-coordinated complexes. Multiple Negishi couplings with C_6_Br_6_ could be performed in a single pot to produce hexakis(ferrocenylethynyl)benzene and similar *π*-conjugated molecules, using excess ZnCl_2_ to prevent formation of homodimers (disubstituted butadiynes) (scheme 34) [119]. Hybrid PAE copolymers with pendant ferrocene units have also been synthesized by Stille polycondensation with molecular weights near 7 kDa (scheme 35) [120]. The thermal stability of these hybrid PAE−fluorenylferrocenes is remarkably high, and they are able to withstand a 30 min exposure at 400 °C. 35

**Scheme 36.**
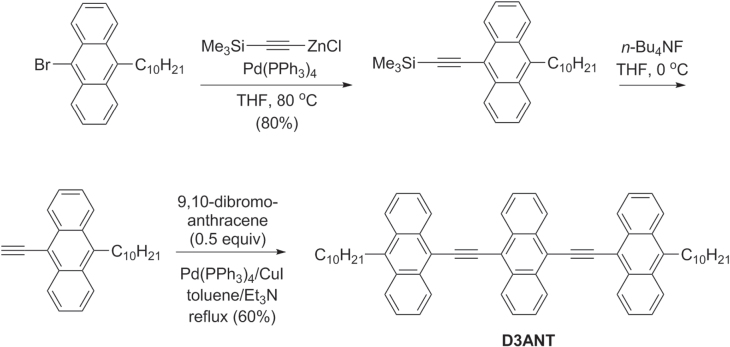
Synthesis of teranthrylethynylene (D3ANT), a candidate material for TFTs.

Oligo(arylethynylenes) are candidate materials for organic TFTs, due to their propensity to adopt planar conformations and form crystalline domains. For example, the molecule 9,10-teranthrylethynylene (D3ANT) was synthesized via Negishi and Sonogashira coupling in good yield (scheme 36), and examined for its thermal and electrochemical stability [121]. Solutions of D3ANT are highly processable and can be cast into top-contact devices to support hole mobilities of 0.012–0.055 cm^2^ V^−1^ s^−1^ with on−off ratios greater than 10^4^, and can self-assemble into highly ordered monolayers when cast onto graphite or Au(111) surfaces.

### 3.5. Oligo- and poly(arylvinylene)s

Polyarylvinylenes (PAVs) represent one of the largest classes of organic semiconductors, and are well known for their high electron affinities and superior electroluminescent properties. The *π*-conjugated backbones of PAVs can support a variety of functional groups for enhanced processability and adjustable reduction potentials; the parent structure, poly(*p*-phenylene)vinylene (PPV), has been extensively modified to produce OLEDs with tunable emissions at visible wavelengths [122, 123]. Here we present some recent examples of PAVs prepared by TM-catalyzed cross-coupling reactions.

**Scheme 37.**
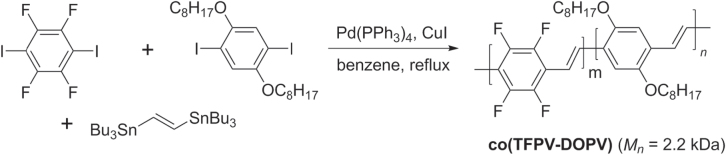
Synthesis of tetrafluorophenylvinyl−dialkoxyphenylvinyl copolymer (co(TFPV-DOPV)) using Stille conditions.

Many PAVs can be prepared by Stille polycondensation, which can couple vinylstannanes with electron-poor or electron-rich components with similar efficiencies. This method has been used to prepare PPV copolymers containing tetrafluorophenylvinyl and 2,5-dialkoxyphenylvinyl units with unusually high nonlinear optical response (scheme 37) [124]. The TFPV−DOPV copolymer is also photoluminescent; when prepared as a thin film, a large Stokes shift in emission wavelength is observed from 540 to 630 nm, indicative of interchain migration.

**Scheme 38.**
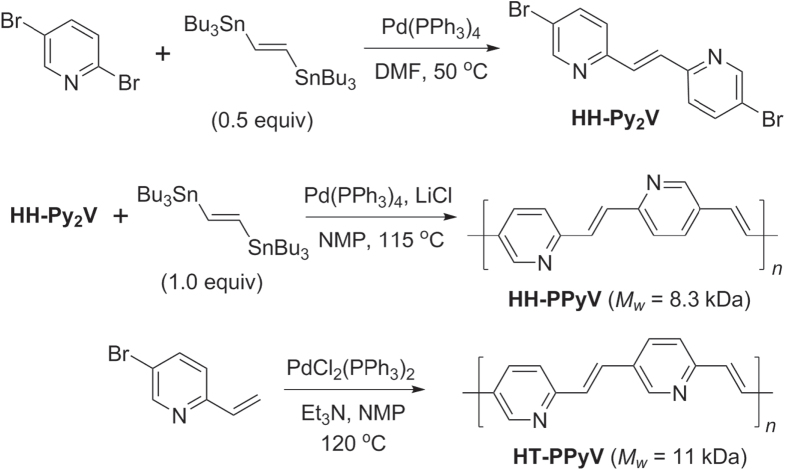
Synthesis of regioregular poly(methylpyridinium vinylene)s (HH- and HT-PMPyVs) by Stille and Heck couplings, respectively. NMP = *N*-methylpyrrolidinone.

Pd-catalyzed cross-couplings are also compatible with coordinative heteroarenes, and their polymerizations can proceed with similar levels of efficiency as with standard aryl subunits. Both Stille and Heck polycondensations have been used to generate various poly(pyridylvinylene)s (PPyV)s, which can serve as electron-deficient PPV analogs [125]. Stille couplings were used to produce HH bis(pyridyl)ethene, which was further condensed with distannylethylene to produce regioregular HH-PPyVs, whereas the polymerization of 5-bromo-2-vinylpyridine using Heck conditions yielded HT-PPyVs (scheme 38). PPyVs have been found to be photoluminescent at 600 nm, with efficiencies of up to 14% [126].

**Scheme 39.**
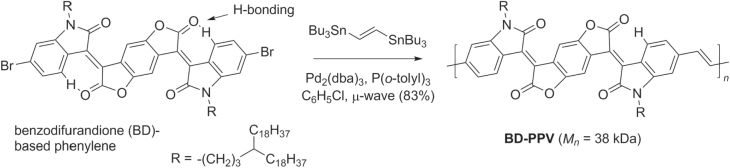
Synthesis of a benzodifurandione-based PPV using Stille coupling.

Stille cross couplings have been used to prepare benzodifurandione-based PPV (BD-PPV) derivatives, electron-deficient analogues designed to address the issue of carrier mobility in solid-state PPVs. Problems associated with low carrier mobility include low crystallinity, *cis/trans* isomerization of the vinylene subunits in individual chains, and high LUMO levels that discourage efficient electron transport [127]. The BD-PPV backbone is more rigid than typical PPVs and is further stabilized by intramolecular CH···O hydrogen bonds (scheme 39), which promotes *π*–*π* stacking between chains. The conformationally locked structure is also stable upon exposure to UV or visible irradiation, and can withstand temperatures up to 390 °C. Electronic characterization of BD-PPV revealed low HOMO and LUMO levels, below that of other *n*-type polymers such as conjugated naphthalene or perylene diimides. BD-PPV can also form highly crystalline domains, and TFT devices could support electron carrier mobilities up to 1.1 cm^2^ V^−1^ s^−1^, over 10^4^ times higher than in standard PPVs.

**Scheme 40.**
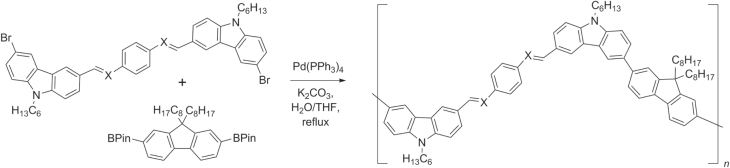
Synthesis of PAV copolymers and their poly(azomethine) analogs by Suzuki polycondensation. Pin = pinacolato.

PAVs and their precursors have also been synthesized by Pd-catalyzed Suzuki and Hiyama couplings, using arylborinates and vinylsiloxanes, respectively. Carbazolyl−fluorenyl-based PPV copolymers and their polyazomethine analogs were prepared by Suzuki couplings with average molecular weights of 5 kDa (scheme 40) [128]. Polyazomethines are well known for their photoconductive properties [129] and are commonly synthesized by the condensation of diamines and dialdehydes; however, this approach may be limited at times by the poor reactivity of either or both components.

**Scheme 41.**
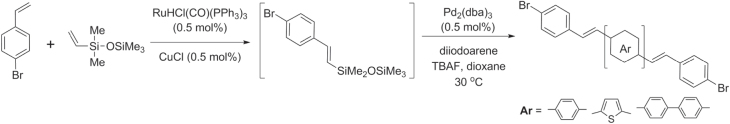
One-pot tandem synthesis of oligophenylvinylenes from 4-bromostyrene, using olefin cross-metathesis and Hiyama coupling; dba = dibenzylideneacetone; TBAF = tetrabutylammonium fluoride.

Hiyama coupling conditions are milder than Suzuki or Stille conditions, and the vinyl- or arylsiloxane building blocks are stable and easy to work with. Moreover, Hiyama conditions can circumvent the use of Brönsted bases, relying instead on fluoride ions for desilylative coupling [130]. For example, oligo(4-halostyryl)arenes have been prepared by a one-pot synthesis using Pd_2_(dba)_3_ and the corresponding styryldisiloxane, which in turn is readily prepared by Ru-catalyzed olefin cross metathesis (scheme 41) [131].

**Scheme 42.**
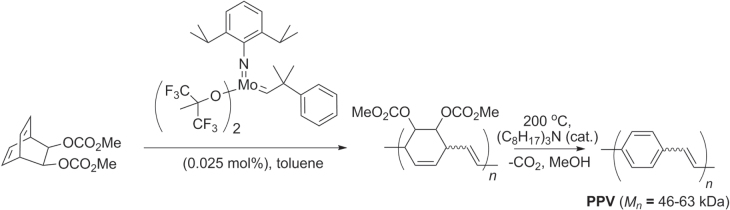
PPV synthesis by ROMP of a disubstituted bicyclooctadiene, followed by thermal elimination.

Finally, olefin metathesis has been used to prepare polymers with alternating cyclohexene−ethylene units, *en route* to PPVs. Ring-opening metathesis polymerization (ROMP) of a disubstituted dicyclooctadiene was performed using Schrock’s catalyst to produce polymers with low polydispersity (PDI = 1.2–1.3, *M*_*n*_ = 46–63 kDa), which could undergo double elimination to produce PPVs with a mixture of *cis* and *trans* alkenes (scheme 42) [132]. It is worth noting that low-molecular weight PPVs have been prepared by ROMP of strained (2.2)paracyclophanedienes and from 1,4-divinylbenzenes [133, 134], but to our knowledge high molecular weight PPVs have not yet been achieved directly from 1,4-divinylbenzenes by olefin metathesis.

## 4. Concluding remarks

Enormous progress has been achieved in the synthesis of organic electronic materials over the past two decades. TM-catalyzed cross-coupling has played a key role in producing numerous types of *π*-conjugated oligomers and polymers, with many appealing properties for practical applications in organic electronics. The main advantages of the synthetic methodologies discussed herein are their compatibility with many types of functional groups and the ease in preparing various monomers, making it possible to synthesize complex polymers with high molecular weight. Polythiophenes, polyfluorenes, polyarylenes, -arylethynylenes, and -arylvinylenes have been produced using TM-catalyzed cross-couplings, some already used in commercial production.

Despite the many significant discoveries and developments in TM-catalyzed cross-coupling, there remain some unsolved problems. The low reactivity of aryl or alkenyl chlorides represents one limitation. The use of chlorides would present a great advantage, considering these substrates are often less expensive and in greater supply than the corresponding bromides. It is also desirable to develop more powerful catalysts, particularly those featuring sterically demanding phosphine or *N*-heterocyclic carbene ligands, to improve the cross-coupling efficiency of challenging substrates. High catalyst loadings present another challenge: palladium catalysts are relatively expensive, and the colloidal palladium formed during polymerization could be detrimental to the electronic materials properties of the resulting polymers if not properly removed. Efforts to improve catalyst efficiency and turnover number are now in development, as recently demonstrated by a PdP(*t*Bu)_3_-catalyzed Negishi polymerization with exceptionally high catalyst turnover numbers (TON > 200 000) [79]. An alternative solution is to recycle and reuse catalysts immobilized on solid support or suspended in separable liquid media (e.g., fluorous or ionic liquid phases).

TM-mediated C–H activation continues to be a promising avenue for polymer synthesis, as it circumvents the use of organic halides and/or the generation of organometallic precursors. This field is still at an early stage of development relative to established TM-catalyzed cross-coupling reactions, but its advancement will likely be influenced by socioeconomical factors and by demonstrations of its practical use on a large scale. In the meantime, it is desirable to identify solutions to issues of chemo- and regioselectivity, which has so far limited the choice of substrates amenable to C–H bond activation. Taking into account the current state of development in TM-catalyzed cross-coupling, we believe that this class of reactions is likely to become increasingly important to the future of organic electronic materials.
